# Treatment Outcome and Prognosis Factors of FIGO 2018 Stage III Cervical Cancer Patients Treated with Definitive Concurrent Chemoradiation in Vietnam

**DOI:** 10.31557/APJCP.2021.22.3.853

**Published:** 2021-03

**Authors:** Huyen Thi Phung, Minh Cong Truong, Long Thanh Nguyen, Anh Thi Van Dang, Thanh Ha Vu, Hoa Thi Nguyen

**Affiliations:** 1 *Department of Medical Oncology 6, Vietnam National Cancer Hospital, Hanoi, Vietnam. *; 2 *Department of Oncology, Vietnam University of Traditional Medicine, Hanoi, Vietnam. *; 3 *Department of Oncology, Hanoi Medical University, Hanoi, Vietnam. *; 4 *Department of Breast and Gynecologic Radiotherapy, Vietnam National Cancer Hospital, Hanoi, Vietnam. *; 5 *Department of Medical Oncology 2, Vietnam National Cancer Hospital, Hanoi, Vietnam. *

**Keywords:** Cervical cancer, stage III, 3D CRT, 3-year DFS, late toxicity

## Abstract

**Objective:**

This study aimed to analyze the treatment outcome and toxicities, along with prognosis factors of patients with FIGO 2018 stage III cervical cancer treated with definitive concurrent chemoradiation.

**Methods:**

A total of 83 stage III cervical cancer patients with good performance status (ECOG PS 0, 1) were treated with three-dimensional conformal radiation therapy (3D-CRT) combined with chemotherapy (weekly cisplatin), followed by high-dose-rate (HDR) brachytherapy between January 2017 and March 2019 at Vietnam National Cancer hospital. Treatment outcomes and prognosis factors were assessed along with acute and late toxicities.

**Results:**

The 3-year DFS was 67.8% and 3-year OS was 80.3%. On multivariate analyses, short axis of pelvic lymph node diameter of ≥ 15mm, invasion of the lower third of vagina and para-aortic lymph node metastasis were identified as adverse prognostic factors for DFS. The cumulative incidence rate of gastrointestinal and genitourinary toxicity (≥ grade 2) at the 3-year follow-up were 29.6% and 11.6%, respectively.

**Conclusions:**

3D CRT and HDR brachytherapy with concurrent chemotherapy is an effective treatment, with acceptable toxicity for FIGO 2018 stage III cervical cancer in Vietnam.

## Introduction

Cervival cancer has still been one of the most common cancers globally, especially in developing countries where HPV vaccination has not been widely implemented (Bray et al., 2013). According to GLOBOCAN 2018, cervical cancer ranked fourth in both incidence and mortality rates among female cancers, with about 8.6 million new cases and 4.2 million dealths each year (Bray et al., 2018). In countries with low-middle Human Development Index, cervical cancer is the second most common cause of malignancy-related mortality and morbidity, and more than 70% patients presented at advanced stages of disease (Gargiulo et al., 2016). In Vietnam, the lack of routine population-based screening and HPV vaccination leads to the majority of patients being diagnosed with locally advanced cervical cancer (Pham et al., 2019).

Currently, early cervical cancer (FIGO stage IA1-IIA1) is usually treated with surgery, but concurrent chemoradiation is the standard of care for locally advanced cases (FIGO stage IB2-IVA). Since early 2000s, American National Cancer Institute has published multiple large-scale randomized clinical trials showing that concurrent chemoradiation reduced the recurrence rate and improved survival compared to radiotherapy alone in locally advanced cervical (Keys et al., 1999; Morris et al., 1999; Rose et al., 1999; Whitney et al., 1999; Peters III et al., 2000).

In 2018, The International Federation of Gynecology and Obstetrics (FIGO) revised their 2014 staging system with significant changes as regional nodal metastasis (pelvic and para-arotic lymph nodes) was classified as stage IIIC regardless of tumor diameter and invasion into surrounding structures (except for bladder and rectum) (Bhatla et al., 2019). As a result, stage III has become the most heterogenous stage, and factors for further risk stratification and prognosis of this stage should be identified (Liu et al., 2019; Grigsby et al., 2020). Besides, to our best knowledge, data on treatment outcome and toxicities in patients with stage III cervivcal cancer according to the new FIGO system is limited, especially in Asian population (Liu et al., 2020; Raut et al., 2020). 

In the present study, we aim to analyze the treatment outcome and toxicities, along with prognosis factors of Vietnamese patients with FIGO 2018 stage III cercical cancer treated with definitive concurrent chemoradiation.

## Materials and Methods

*Study design*

This was a retrospective cohort study of patients with stage III cervical cancer treated at Vietnam National Cancer Hospital. Our institution is a referral oncology center for Northern Vietnam and is among few centers that provide radiotherapy for cervical cancer in the country. Convenience sampling method was used to enroll patients admitted from January 2017 to March 2019. The protocol of this study was approved by the human research ethics committee at Vietnam National Cancer Hospital.

*Study population*

*Inclusion and exclusion criteria*

The eligibility criteria included cervical cancer diagnosed by histopathology of biopsy specimen; treated with definitive concurrent chemoradiation; an Eastern Cooperative Oncology Group performance status score less than 2; and whose medical records and radiation data were available to the investigators. The stage was assigned in accordance with the 2018 FIGO Staging System for Uterine Cervical Cancer (Bhatla et al., 2019). Pelvic/para-aortic lymph nodes involvement was defined as one of the following: (1) having FDG accumulation greater than liver accumulation or standard uptake value (SUV)> 2.5 ng/ml on positron emission tomography/computed tomography (PET/CT), or (2) the short axis diameter ≥ 10mm on pelvic magnetic resonance imaging (MRI) or CT (Liu et al., 2019). We excluded patients who underwent palliative RT only or previous treatment for cervical cancer, those who received preoperative chemoradiation followed by surgery, those with neuroendocrine carcinoma of the cervix and patients who did not had either abdominopelvic CT scan or pelvic MRI for lymph node assessment.

*Data* *collection*

We retrospectively reviewed medical reports of patients treated with concurrent chemoradiation at our institution during the study period. Patients assessed as stage III cervical cancer according to the former FIGO 2014 staging system and those with pelvic/para-aortic lymph node metastasis on imaging that satisfied study criteria were selected. Data on patients’ demographic, tumor, lymph nodes characteristics and toxicities along with disease free survival (DFS) and overall survival (OS) was recorded. 

*Treatment Protocol and Follow-Up*

*Radiation therapy*

The patients were treated with three-dimensional conformal radiation therapy (3D CRT) combined with chemotherapy, followed by high-dose-rate (HDR) brachytherapy. The clinical target volume (CTV) included the gross tumor volume (GTV), cervix, entire uterus, parametrium and upper part of the vagina to 3 cm below the most inferior extent of tumor. The CTV-node included the common, external, and internal iliac and presacral lymph nodes and/or para-aortic lymph nodes with a 7 mm margin around the vessels and any additional visible lymph nodes. The planning target volume (PTV) was created by expanding the CTV with a 10 mm margin and an additional 5 mm margin to the cervix and uterus. Patients were then treated with anterior (AP), posterior (PA) and lateral conformal fields with daily dose of 1.8 Gy - 2 Gy to a total dose of 45- 50.4 Gy. Bladder and rectal blocks after 36-40Gy and an additional boost to the parametrium or metastatic lymph nodes to 59-61 Gy were given at the discretion of the treating physician. 

A dose of 30-36 Gy in 3-5 fractions was delivered to point A in patients receiving 2D brachytherapy according to International Commission of Radiation Units (ICRU) 38. For patients who underwent 3D brachytherapy, at least 90% of the high-risk CTV received a dose of 30-36 Gy in 3-5 fractions. 

*Chemotherapy*

The standard chemotherapy regimen was weekly cisplatin (40 mg/m^2^) for 5 cycles during the external beam phase of the radiation therapy. Chemotherapy and radiation therapy were started simultaneously. 

*Toxicities*

The early and late toxicities were assessed during treatment and at each 2-3 month-follow-up according to RTOG/EORTC (Radiation Therapy Oncology Group and European Organization for Research and Treatment of Cancer) scale (Cox, 1995).

*Data analysis*

Data was analysed using IBM SPSS Statistics for Windows version 26.0 (IBM Corp., Armonk, NY). Depending on the distribution, continuous data were presented as mean±standard deviation or median (interquartile range (IQR)) and categorical data as number (percentage). Comparison was done using the Fisher’s exact test and Chi-squared tests as appropriated. OS and DFS were estimated by the Kaplan–Meier method and compared by the log-rank test. Stepwise forward direction selection strategy was used to identify parameters for multivariate Cox proportional hazard regression models to evaluate factors associated with DFS. Factors and cut-off selected in the survival analysis were selected based on literature review (Song et al., 2013; Liu et al., 2019). All tests were two-tailed and differences were considered statistically significant at p values <0.05.

## Results

*Patients characteristics*

A total of 83 patients with stage III cervical cancer were treated with definitive concurrent chemoradiation in our institution from January 2017 to March 2019. The study population had a median age of 55 (IQR 46-61), most of which had squamous cell carcinoma (92.8%). The mean tumor diameter was 50.8±15.8 mm. Details on tumor invasion and nodal metastasis were presented in Table 1. Most patients had stage IIIB or IIIC1 according to FIGO 2018 classification due to pelvic wall invasion and pelvic lymph node metastasis (34.9% and 60.2% respectively).

Nearly all patients (99%) completed the treatment, in which the majority had an A point dose of 80-90Gy and received ≥4 cisplatin cycles (accounting for 68.7% and 80.7% respectively). Almost half of the cases finished treatment within 8 weeks. Beside 3 patients with para-aortic lymph node metastasis, 13 other patients who were considered high risk for para-aortic lymph node metastasis by treating clinician also received extended-field radiotherapy.

*Outcome and pattern of recurrence*

The median duration of follow up was 29.6 months (range 4.0 – 44.5 months), disease recurrence or death was recorded in 23 (27.7%) patients. The 3-year DFS was 67.8% and 3-year OS was 80.3%. The 3-year DFS for stage IIIB and IIIC1 were 70.8% and 69.7% respectively. Univariate analysis results indicated that lower third vaginal involvement, short axis diameter of pelvic nodes ≥ 15 mm, para-aortic lymph node metastasis and total treatment duration > 8 weeks were associated with disease recurrence. Meanwhile, multivatiate analysis with stepwise approach only identified the former three features were significant prognostic factors for reccurence (see Table 3 for details).

Among cases with recurrence at primary tumor site, 6/7 patients had bulky tumor (> 4 cm in diameter) and 3/7 patients had lower third vaginal involvement. Meanwhile, 3/6 pelvic node recurrences had short axis diameter of pelvic nodes ≥ 15mm at presentation. There were 3 cases with para-aortic recurrence, two of which were in-field of EFRT radiotherapy treatment.

*Toxicities*

The numbers of patients with acute and late toxicities from chemoradiation treatment were presented in Table 5. During treatment, 31 (37.3%) patients had neutropenia and 4 (4.8%) patients had thrombocytopenia. Grade 3-4 hematological toxicities were recorded in 7 (8.4%) cases. The cumulative incidence rate of gastrointestinal and genitourinary toxicity (≥ grade 2) at the 3-year follow-up was 29.6% and 11.6%, respectively. 

**Table 1:** Patient Demographics, Clinical and Imaging Characteristics

Characteristic	No. of patients (%) N=83
Age (median (IQR))	55 (46-61)
Histology	
SCC	77 (92.8%)
Adenocarcinoma	6 (7.2%)
Tumor size	
≤ 40mm	23 (27.7%)
> 40mm	60 (72.3%)
Vaginal involvement	
No involvement	3 (3.6%)
Upper third	57 (68.7%)
Middle third	13 (15.7%)
Lower third	10 (12.0%)
Parametrial infiltration	
No involvement	13 (15.7%)
Unilateral	19 (22.9%)
Bilateral	51 (61.4%)
Invasion of pelvic wall	
No	40 (48.2%)
Yes	43 (51.8%)
Pelvic lymph node metastasis	
No	29 (34.9%)
Unilateral	28 (33.7%)
Bilateral	26 (31.3%)
Short axis diameter of pelvic lymph nodes	
< 15mm	65 (78.3%)
≥ 15mm	18 (21.7%)
Para-aortic lymph node metastasis	
No	80 (96.4%)
Yes	3 (3.6%)
Stage (FIGO 2018)	
IIIA	1 (1.2%)
IIIB	29 (34.9%)
IIIC1	50 (60.2%)
IIIC2	3 (3.6%)

**Table 2:** Radiotherapy and Chemotherapy Treatment Characteristics

Characteristic	No. of patients (%) N=83
Extended field radiotherapy (EFRT)	
Yes	16 (19.3%)
No	67 (80.7%)
Total A point dose	
< 80 Gy	19 (22.9%)
80-90 Gy	57 (68.7%)
> 90 Gy	7 (8.4%)
Completion of radiotherapy	
Yes	82 (98.8%)
No	1 (1.2%)
Duration of radiotherapy	
≤8 weeks	41 (49.4%)
>8 weeks	42 (50.6%)
Concurrent chemotherapy	
1-3 cycles	16 (19.3%)
≥4 cycles	67 (80.7%)

**Figure 1:**
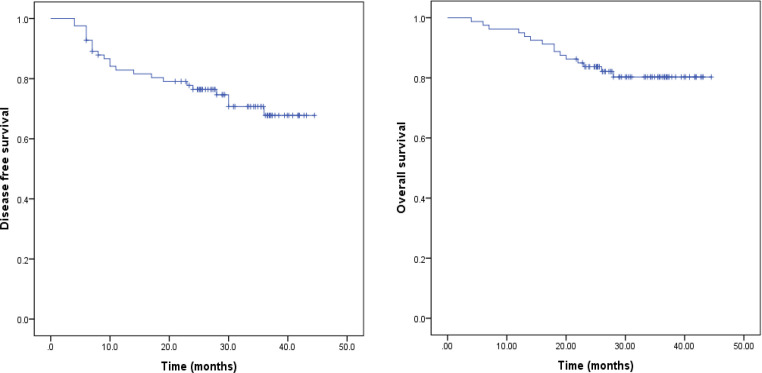
Kaplan–Meier Curve of Disease-Free Survival (DFS) and Overall Survival (OS) of Study Population

**Table 3:** Factors Associated with 3-Year Disease-Free Survival (DFS) in Univeariate and Multivariate Cox Proportional Hazards Model

Factors	N=83 (%)	3-year DFS	Univariate analysis	Multivariate analysis	
			p	HR (95% CI)*	p
Histology					
SCC	77	68.00%	0.749	-	-
AC	6	62.50%			
Tumor size					
≤ 40mm	23	81.80%	0.106	-	-
> 40mm	60	63.00%			
Lower third vaginal involvement					
No	73	74.10%	<0.001	12.373	4.399-34.806
Yes	10	17.50%			
Parametrial infiltration					
No	13	76.20%	0.746	-	-
Yes	70	67.10%			
Pelvic wall invasion					
No	40	73.20%	0.501	-	-
Yes	43	63.20%			
Bilateral nodal metastasis					
No	57	72.10%	0.182	-	-
Yes	26	53.80%			
Short axis diameter of pelvic nodes					
< 15mm	65	74.00%	0.008	4.07	1.666-9.943
≥ 15mm	18	45.80%			
Para-aortic lymph node metastasis					
No	80	70.40%	<0.001	21.095	5.357-83.068
Yes	3	0%			
Treatment duration					
≤8 weeks	41	84.90%	0.017	-	-
>8 weeks	42	55.80%			

*, Hazzard ratio of recurrence

**Table 4:** Pattern of Relapse after Definitive Concurrent Chemoradiation*

Stage	Pelvic		Para-aortic	Distant	Unknown**
	Central	Nodal			
IIIA	-	-	-	-	-
IIIB	5	2	1	4	-
IIIC1	2	4	2	5	2
IIIC2	-	-	-	-	3
Total	7	6	3	9	5

*, More than one sites of relapse could be recorded in one patient; **, Death without any documented recurrence sites.

**Table 5:** Incidence of Treatment-Related Acute Hematologic Toxicity (%)

Toxicity	Grade 1	Grade 2	Grade 3	Grade 4
Acute toxicities				
Neutropenia	19 (22.9%)	12 (14.5%)	6 (7.2%)	1 (1.2%)
Thrombocytopenia	4 (4.8%)	0	0	0
Late toxicities				
Gastrointestinal	20 (24.1%)	13 (15.7%)	7 (8.4%)	2 (2.4%)
Genitourinary	9 (10.8%)	6 (7.2%)	1 (1.2%)	1 (1.2%)

## Discussion

Since the introduction of the new FIGO classification in 2018, there have been significant changes in cervical cancer staging in clinical practice and stage III has been more commonly encountered at disease presentation. In a retrospective validation study on 1,282 patients from 1997 to 2019, while FIGO 2018 had better survival discriminatory ability compared to FIGO 2009, survival of stage III subclassifications were heterogeneous (Grigsby et al., 2020). 5-year PFS of stage IIIA, IIIB, IIIC1 and IIIC2 were 46%, 55%, 62%, 35% respectively, in which the prognosis of patients with stage IIIC1 was even better than those with stage IIIB (Grigsby et al., 2020). In our study, most patients had FIGO 2018 stage IIIB or IIIC1 (accounted for 34.9% and 60% respectively), both groups had approximately equal 3-year DFS of about 70%. However, one should note that 24% of our patients with stage IIIC1 had T3b disease (pelvic wall invasion), which might contribute to the attenuated difference between the two substages. Another validation study on Asian cohort reported similar 3-year DFS rates of 70.4% for IIIB and 66.3% for stage IIIC1 (Liu et al., 2020). Compared to stage IIIC1, stage IIIC2 had significantly poorer outcomes. In an analysis of 17,173 patients based on National Cancer Database, para-aortic lymph node metastasis was associated with a hazard ratio 2.14, p<0.001 for overall survival in multivariate analysis, with 5-year OS of only 39.4% (McComas et al., 2020) . In our study, all three patients with stage IIIC2 have died, in which the longest survival was 19 months.

A large-scaled analysis of The Surveillance, Epidemiology, and End Results Program data on 11,733 patients with stage III cervical cancer showed that 5-year cause-specific survival of stage IIIC1 was significantly associated with T stage (74.8% for T1, 58.7% for T2, and 39.3% for T3) (Matsuo et al., 2019). Therefore, besides FIGO stage, the detail extent of tumor invasion also plays an important role in disease prognosis and might provide further risk stratification. In our study, lower third vaginal involvement (LTI) was an independent risk factor for disease recurrence in multivariate regression, regardless of nodal involvement and pelvic wall invasion. In a retrospective matched analysis of 216 patients with stage IIIB cervical cancer, LTI was associated with inferior PFS and OS, with hazard ratios of 1.64 (95%CI 1.12–2.41) and 1.63 (95%CI 1.11–2.40) respectively (Katanyoo, 2017). In this study, patients with LTI had higher risk of local progression but not distant metastasis, probably due to inadequate local treatment since the lower part of the vagina might not be fully reached by brachytherapy. 

Beside LTI, large nodal disease (short axis diameter of pelvic lymph nodes ≥ 15mm) and para-aortic lymph node metastasis were also associated with recurrence in our cohort. In the group with large nodal metastasis, 9/18 patients recurred (5.07 times higher chances compared to those without), in which 5/9 cases developed recurrent pelvic/para-aortic nodes. The impact of not only the presence of lymph node metastasis but also the characteristics of the nodes on survival for patients with cervical cancer has been investigated in several studies (Narayan et al., 2009; Song et al., 2013; Li et al., 2016). Song et al reported that the 5-year DFS for patients with negative lymph node, lymph node diameter <15 mm and lymph node diameter ≥15 mm were 80%, 67% and 50%, respectively (P < 0.001) (Song et al., 2013). The size of lymph node also significantly affected survival in a study of Li et, in which the 3-year OS of patients with lymph node ≥15mm in size was 72.1%, significantly lower than 87.1% in patients with lymph node <15mm (Li et al., 2016). Moreover, the number and volume of pelvic lymph node metastasis, matted/necrotic lymph nodes were also associated with survival in patients with cervical cancer (Li et al., 2016; Liu et al., 2019). In patients with high-risk lymph node characteristics, lymph node–directed simultaneous integrated boost with intensity-modulated radiation therapy (IMRT) or Volumetric Modulated Arc Therapy (VMAT) might be beneficial to improve locoregional control with acceptable rates of adverse events (Jethwa et al., 2018). Our study employed 3D conformal radiation therapy, which did not facilitate simultaneous lymph node boost and the in-field lymph node recurrence rate was considerably high in paitents with large pelvic node metastasis. In a developing country like Vietnam, inadequate insurance coverage and waiting list issue due to shortage of modern radiotherapy facilities might put the use of IMRT/VMAT for cervical cancer under careful consideration. In a recent report of International Atomic Energy Agency (IAEA), out of pocket health expenditure was considerably high in Southeast Asia, accounting for 53.95%, 40.47% and 55.42% of total health expenditure in Philippines, Malaysia and Singapore respectively, whereas a significant proportion of radiotherapy services are owned by private sector in these countries (Rosenblatt and Zubizarreta, 2017). The results of this study can help physician to identify the subgroups of patients who may have more benefits from modern and more expensive radiation therapy techniques. Apart from simultaneous boost, extended-field irradiation therapy (EFRT) should also be considered in patients with lymph node diameter ≥15 mm, according to a recent report of Wang et al (Wang et al., 2019). Adding more chemotherapy is an alternative approach of escalated treatment for high risk patients. A phase III study comparing cisplatin plus gemcitabine during concurrent RT plus two adjuvant cycles of cisplatin-gemcitabine with cisplatin alone for patients with locally advanced cervical cancer demonstrated improvement in DFS and OS but was associated with significantly more serious (grade 3/4) toxicities (Dueñas-González et al., 2011). We are still awaiting the results of some ongoing trials such as OUTBACK (ClinicalTrials.gov identifier: NCT01414608) and INTERLACE (ClinicalTrials.gov identifier: NCT01566240) trials which investigate adjuvant and neoadjuvant taxane-platinum chemotherapy added to concurrent chemoradiation. These trials will provide further evidence for the escalated chemotherapy treatment and might bring new therapeutic options for high-risk cervical cancer.

As discussed above, the presence of locoregional nodal disease is an important prognosis factor and might play a role in treatment planning. Pre-treatment nodal evaluation by abdominopelvic CT scan in locally advanced cervical cancer is necessary even in developing coutries, as it could help to avoid unnecessary surgery and might be cost-effective (Haldorsen et al., 2019). However, there is still some debates in the size threshold to define lymph node positivity on imaging, and the most widely criterion is the short axis diameter ≥ 10 mm (Huang and Fang, 2018). Although smaller lymph node might habor metastatic tumor, a previous study suggested that failure in pelvic lymph nodes is rare in cases with nodes smaller than 10 mm in short diameter (Song et al., 2013).

In our study, a treatment duration over 56 days also influenced treatment outcome in univariate analysis for DFS. Several studies have recorded that treatment time exceeding 8 weeks and nonoptimal RT had poorer survival (Diaz et al., 2014; Ohri et al., 2016). However, in the stepwise multivariate analysis, treatment time longer than 8 weeks was not significantly associated with inferior outcome, probably because most patients with treatment time over 56 days had more fractions of brachytherapy rather than RT noncompliance, similar to the findings of Katanyoo (Katanyoo, 2017). 

In terms of toxicities, the 3-year cumulative incidence rates of gastrointestinal (GI) and genitourinary (GU) toxicity (≥ grade 2) were 29.6% and 11.6%, respectively. Late GI and GU toxicities of grade 3, 4 included rectal bleeding that required surgery (7 cases), rectovaginal fistula (1 case), small bowel obstruction (1 case) and hematuria requiring cystoscopy (2 cases). Our rates are comparable to the data reported in a systematic review of 19 trials of concurrent chemoradiation with 3D-CRT (Kirwan et al., 2003). In which, the rates of grade 1-2 and 3-4 of GI and GU toxicities were 45.2%, 17.5%, 8%, 1.5% respectively (Kirwan et al., 2003). Chronic toxicities were considerably lower in a recent report in China in which IMRT was ultilized in the majority of patients (Wang et al., 2017). Several studies have shown benefits of IMRT/VMAT in terms of reduction in dose to organs at-risk and toxicities (Yahya et al., 2015; Lei et al., 2019). However, further studies are required to robustly confirm the superiority of IMRT/VMAT over 3D-CRT for cervical cancer.

Our study has some limitations: the study is a retrospective single institution analysis, with small sample size and convenience sampling method. Besides, since we only included patients who had abdominopelvic CT scan or pelvic MRI for lymph node assessment, our study is subjected to selection bias. Moreover, due to its retrospective nature, more detailed information such as number and location (common versus external/internal iliac) lymph nodes could not be fully recorded. 

In consluion, 3D CRT and HDR brachytherapy with concurrent chemotherapy is an effective treatment, with acceptable toxicity, for FIGO 2018 stage III cervical cancer in Vietnam. Escalated treatment for high risk patients such as those with short axis of pelvic lymph node diameter of ≥ 15 mm, invasion of the lower third of vagina and para-aortic lymph node metastasis should be evaluated in further studies.

## Author Contribution Statement

Conception and design: Phung Thi Huyen, Truong Cong Minh, Nguyen Thanh Long. Administrative support: Phung Thi Huyen, Nguyen Thi Hoa. Collection and assembly of data: Phung Thi Huyen, Truong Cong Minh, Nguyen Thanh Long, Dang Thi Van Anh, Vu Ha Thanh. Data analysis and interpretation: Phung Thi Huyen, Truong Cong Minh, Nguyen Thanh Long, Nguyen Thi Hoa. Manuscript writing: All authors.Final approval of manuscript: All authors. Accountable for all aspects of the work: All authors. 
